# COMMD proteins function and their regulating roles in tumors

**DOI:** 10.3389/fonc.2023.1067234

**Published:** 2023-01-23

**Authors:** Guangqiang You, Chen Zhou, Lei Wang, Zefeng Liu, He Fang, Xiaoxao Yao, Xuewen Zhang

**Affiliations:** ^1^ Department of Hepatobiliary and Pancreatic Surgery, Second Affiliated Hospital of Jilin University, Jilin University, Changchun, China; ^2^ Department of General Affairs, First Hospital of Jilin University (the Eastern Division), Jilin University, Changchun, China; ^3^ Department of Pediatric Neurology, First Hospital of Jilin University, Jilin University, Changchun, China

**Keywords:** COMMD, cancer, copper homeostasis, endosomal sorting, ion transport, NF-κB

## Abstract

The COMMD proteins are a highly conserved protein family with ten members that play a crucial role in a variety of biological activities, including copper metabolism, endosomal sorting, ion transport, and other processes. Recent research have demonstrated that the COMMD proteins are closely associated with a wide range of disorders, such as hepatitis, myocardial ischemia, cerebral ischemia, HIV infection, and cancer. Among these, the role of COMMD proteins in tumors has been thoroughly explored; they promote or inhibit cancers such as lung cancer, liver cancer, gastric cancer, and prostate cancer. COMMD proteins can influence tumor proliferation, invasion, metastasis, and tumor angiogenesis, which are strongly related to the prognosis of tumors and are possible therapeutic targets for treating tumors. In terms of molecular mechanism, COMMD proteins in tumor cells regulate the oncogenes of NF-κB, HIF, c-MYC, and others, and are related to signaling pathways including apoptosis, autophagy, and ferroptosis. For the clinical diagnosis and therapy of malignancies, additional research into the involvement of COMMD proteins in cancer is beneficial.

## 1 Introduction

**Figure 1:**
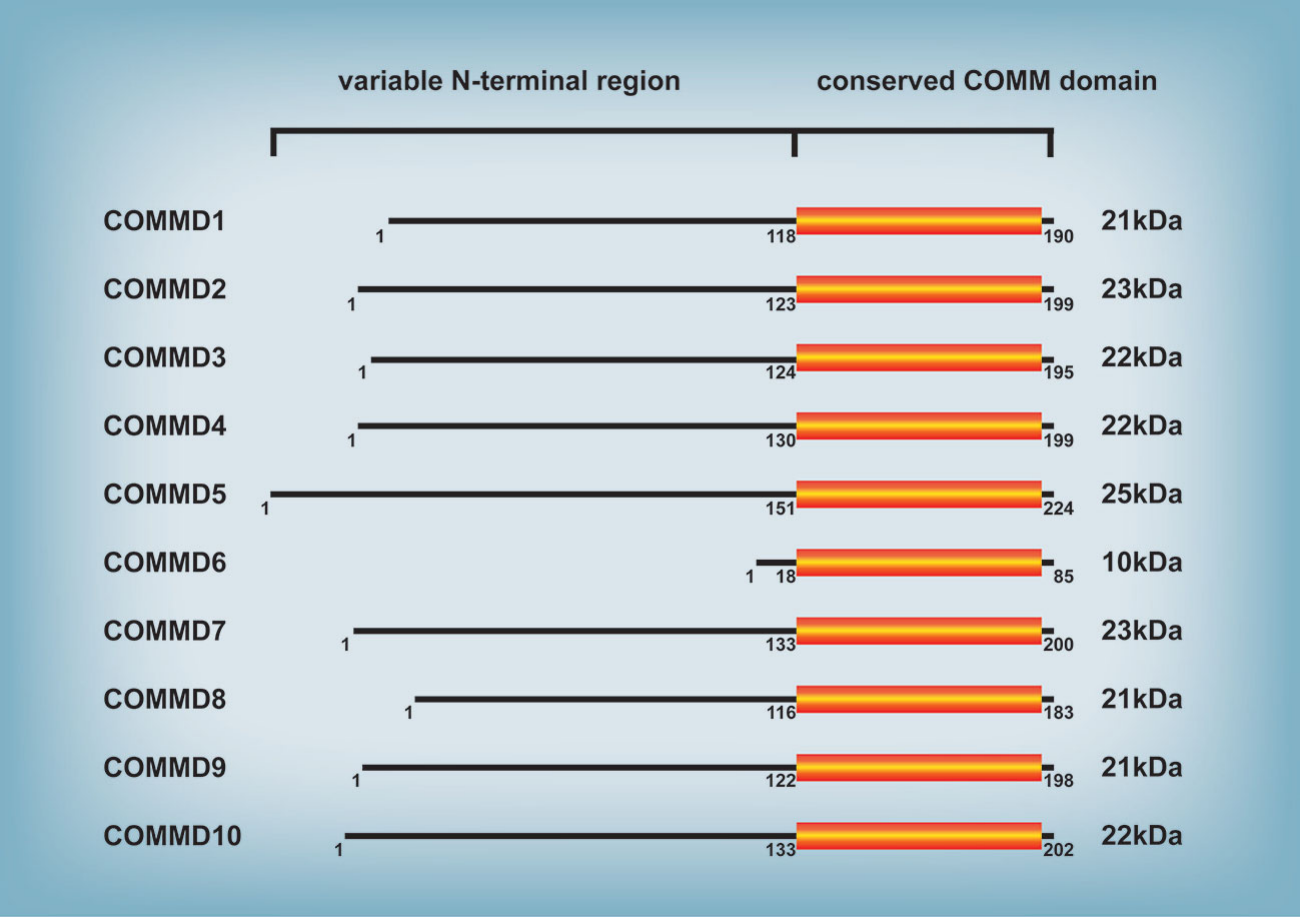
COMMD family of proteins. All ten members of the COMMD protein family share a conserved COMM domain. In contrast, the amino terminal domain is highly variable among different COMMD proteins.

The protein family of copper metabolism MURR1 domain-containing (COMMD) is highly conserved in eukaryotic multicellular organisms and participates in numerous cellular processes, such as copper metabolism, endosomal sorting, ion transport, and transcription factor regulation (1). The COMMD family consists of ten members (**Figure 1**), of which COMMD1 (also known as MURR1) has been the subject of most research since its discovery by Nabetani et al. (2). Exon 2 deletion of the COMMD1 gene was identified in Bedlington Terriers with copper toxicosis in a pervious study (3), demonstrating for the first time that COMMD1 regulates copper metabolism. Copper toxicosis is an autosomal recessive illness characterized by copper secretory abnormalities and hepatic copper accumulation, hepatitis, and even cirrhosis. Subsequently, additional members of the COMMD protein family with comparable structure and a highly conserved COMM domain at the carboxy-terminal region were discovered; the conserved COMM domain participates in protein-protein interaction and nuclear transport, and is ubiquitination targets (4, 5). In contrast, the amino terminal domain is highly variable and practically nonexistent in COMMD6 (4). COMMD proteins are extensively expressed in a range of human tissues, with expression difference between each COMMD member (6) indicating different roles of each COMMD member in particular tissues. In recent years, the majority of research on the function of COMMD proteins has been on COMMD1; little is known about the function of the other COMMD proteins (7). This review focuses on the biological roles of the COMMD protein family and the particularity of COMMD1, and involves as far as possible the research results about other members of the COMMD protein family. Given the functional diversity of COMMD proteins, the COMMD protein family is implicated in numerous disease processes, such as hepatitis (8, 9), myocardial ischemia (10–12), cerebral ischemia (13), HIV infection (14, 15), and malignancies (4). COMMD1 deficiency induces or worsens hepatitis in mouse and dogs, and the mechanism might involve an increase in intracellular hepatic copper and hepatic lipid accumulation (8, 9). Heart ischemia is frequently accompanied by Cu depletion (16). Cu concentration is decreased in the ischemic zone of a mouse model of myocardial ischemia, but COMMD1 protein levels are up. COMMD1-knockdown can decrease the outflow of myocardial Cu, safeguard myocardial function, and diminish the size of infarct focus. However, the cause of COMMD1 rise due to myocardial ischemia requires additional study (10, 12). Although the majority of molecular mechanism research have focused on COMMD1, nearly all COMMD protein family members regulate tumor progression. For example, COMMD1 (17), COMMD4 (18), COMMD8 (19), and COMMD9 (20) regulate the proliferation, invasion, and metastasis of lung cancer. COMMD2 (21), COMMD3 (22), COMMD7 (23), COMMD8 (24), and COMMD10 (25) promote or inhibit hepatocellular carcinoma (HCC). Additionally, COMMD1 (26) and COMMD10 (27) are linked to drug resistance in tumors. After briefly explaining the biological functions of the COMMD protein family, this review then comprehensively detailed the specific roles of COMMD proteins in tumors of diverse systems in order to serve as a resource for future tumor research and clinical diagnosis and therapy.

## 2 The primary biological functions of COMMD protein family

### 2.1 Regulating copper metabolism

Copper is an important trace element that plays a significant role in many biological processes, including cell respiration, cell proliferation, antioxidant defense, neurotransmitter synthesis and angiogenesis. Wilson’s disease, Alzheimer’s disease, and Parkinson’s disease have been associated with an abnormal copper metabolism (28, 29). Normal life activities rely heavily on copper homeostasis being maintained. The liver plays a crucial role in controlling systemic copper homeostasis. Copper is absorbed in the intestine to fulfill the body’s needs, and excreted through the bile to prevent toxicity. Cu transporting ATPase B (Atp7b) in hepatocytes assists secreting excess copper, which will be excreted with bile (28). In humans, mutations in the Atp7b gene are responsible for Wilson’s disease, a disorder that produces hepatic copper overload comparable to copper toxicosis in dogs. Exon 2 deletion of *COMMD1* in Bedlington Terriers results in the loss of COMMD1 protein expression, which ultimately results in aberrant biliary copper excretion and copper toxicosis (30). However, the mechanism by which COMMD1 regulates biliary copper excretion remains unknown. Studies have demonstrated that COMMD1 could bind to mutant Atp7b and promoted its degradation *via* the proteasomal pathway (31). Researchers speculated that COMMD1 is involved in the quality regulation of Atp7b through hydrolyzing misfolded and dysfunctional proteins caused by mutations. Failure to properly regulate Atp7b in Bedlington Terriers due to COMMD1 deficiency may be the underlying cause of copper toxicosis (32). Copper is efficiently secreted due to the bidirectional translocation of Atp7b between trans-Golgi network and cytosolic vesicles. Miyayama et al. discovered that *COMMD1*-knockdown decreased the expression of Atp7b; concurrently, the recycling of Atp7b from cytosolic vesicles to the trans-Golgi network is hindered, resulting in the accumulation of intracellular copper (33). The majority of dietary copper is absorbed in the small intestine, and Atp7a transports copper from small intestine cells to the portal vein. A mutation in the Atp7a gene could cause Menkes’ disease, which is mostly characterized by copper deficiency (28). COMMD1 could bind to both wild-type and mutant Atp7a, increasing Atp7a’s stability and ameliorating the copper exporting capacities of Atp7a mutants (34). In contrast, another research published in the same year shown that COMMD1 triggered the degradation of wild-type and mutant Atp7a through proteasomal pathway (31). The role of COMMD1 in the stability of Atp7a requires additional study. COMMD1 can also regulate the intracellular transport of Atp7a. COMMD1 forms the COMMD/CCDC22/CCDC93 (CCC) complex with CCDC22, CCDC93, and C16orf62, which is necessary for the proper sorting and relocalization of Atp7a (4, 35). COMMD1 can directly bind copper in addition to interacting with Atp7a and Atp7b. However, the importance of this binding effect on copper homeostasis is unexplored (36). There is no indication that *COMMD1* gene mutation induces human copper metabolic-related disorders such as Wilson’s disease, while numerous pieces of evidence indicate that COMMD1 is involved in the regulation of copper metabolism. Also, the role of COMMD2-10 in regulating copper metabolism is rarely explored. It has been observed that the deletion of any one of COMMD1, COMMD6, or COMMD9 leads to a deficiency in the transport of Atp7b from cytosolic vesicles to the plasma membrane in mouse hepatocytes, and a hepatic copper accumulation in conditions of a high-copper diet (37). The function of the COMMD protein family in copper homeostasis requires additional investigation.

### 2.2 Assisting in endosomal sorting of transmembrane receptors

Receptor endocytosis and subsequent sorting is an important way to maintain cell homeostasis. Early endosomes internalize transmembrane proteins and their associated macromolecules, some of which degrade through the lysosomal pathway while others are transported to trans-Golgi network or plasma membrane for reuse by retromer, retriever, WASH complex, CCC complex, and SNX proteins (38). Phosphatidylinositol 4, 5-bisphosphate (PtdIns (4, 5) P_2_) is an important membrane-anchoring molecule, functioning in vesicular trafficking and modulation of transporter activity. The specific binding of COMMD1 to PtdIns(4, 5)P_2_ makes COMMD1 recruited to the endocytotic membranes, where it functions in endosomal sorting (39). Subsequent investigations demonstrated that COMMD1’s endosomal sorting role is performed through the formation of the CCC complex. Depletion of COMMD1 or CCC complex inhibits the transfer of Atp7a from endosomal vesicles to the plasma membrane. CCC complex binds to the WASH complex member FAM21, and CCC complex, WASH complex, and retromer are all implicated in the regulation of endosomal trafficking of Atp7a (35). The low-density lipoprotein receptor (LDLR) plays significant role in eliminating low-density lipoprotein (LDL). CCC and WASH complexes are both involved in the endosomal sorting of LDLR to maintain the homeostasis of circulating cholesterol. Similar to COMMD1, *COMMD6*-knockout and COMMD9 in mouse hepatocytes also destabilizes the CCC complex, resulting in reduced LDLR levels on the cell surface and elevated plasma cholesterol (40, 41). Another research conducted in the same year indicated that COMMD9 is also a component of the CCC complex and is essential for the endosomal sorting of Notch receptors. Given that almost all members of the COMMD protein family can connect with the CCC complex component CCDC22 (40), it is hypothesized that the various COMMD proteins associated with the CCC complex aid in the specific cargos selection (42). Unlike Atp7a, the endosomal cargo recycling of α_5_β_1_-integrin is retromer-independent. Retriever interacts with cargo adaptor SNX17 and combines CCC and WASH complex to facilitate cell surface recycling of α_5_β_1_-integrin (43). In conclusion, CCC complex and related proteins play an important role in the endosomal sorting of transmembrane receptors.

### 2.3 Regulating ion transport

ENaC is an amiloride-sensitive Na^+^ channel, and is comprised of three homologous subunits (α, β, and γ; or δ, β, and γ), which plays a significant role in Na^+^ and body fluid volume homeostasis (44). COMMD1 interacts with Nedd4-2 to increase the ubiquitination and promote the degradation of ENaC (α, β, and γ) (45). In addition, COMMD1 was also found able to bind to δENaC, therefore promoting δENaC into an intracellular recycling pool and reducing δENaC on the cell surface while enhancing ENaC ubiquitination. However, it is unknown if Nedd4-2 is involved in the regulation of δENaC (46). Additionally, COMMD2-10 interacts with ENaC, and later investigations on COMMD3 and COMMD9 have shown that they both lower ENaC expression on the cell surface and amiloride-sensitive current in mammalian epithelia. The COMMD protein family appears to play a negative regulatory role on ENaC (47). However, *COMMD10*-knockdown in Fischer rat thyroid epithelia resulted in increased Nedd4-2 and decreased ENaC current. In conclusion, COMMD1 inhibits Na^+^ transport by promoting ENaC ubiquitination and endocytosis; nevertheless, the regulatory mechanism of COMMD on ENaC proteins and its subunits need more investigation. In epithelial tissue, the cystic fibrosis transmembrane conductance regulator (CFTR) is a vast polytopic cAMP-regulated Cl^-^ channel. Overexpression of *COMMD1* in Hela cells increased the amount of CFTR on the cell surface, while *COMMD1*-knockdown induced ubiquitination and probably proteasomal degradation of CFTR (48). The Na-K-Cl co-transporter 1(NKCC1), a member of the cation-Cl cotransport family, interacts with COMMD1 to ubiquitinate itself, hence enhancing its own expression in the basolateral membrane. One potential reason is that COMMD1 improves the biological activity of NKCC1 by promoting non-classical ubiquitination, which stabilizes rather than degrades the target protein (4, 49).

### 2.4 Regulating NF-κB-mediated transcription

**Figure 2:**
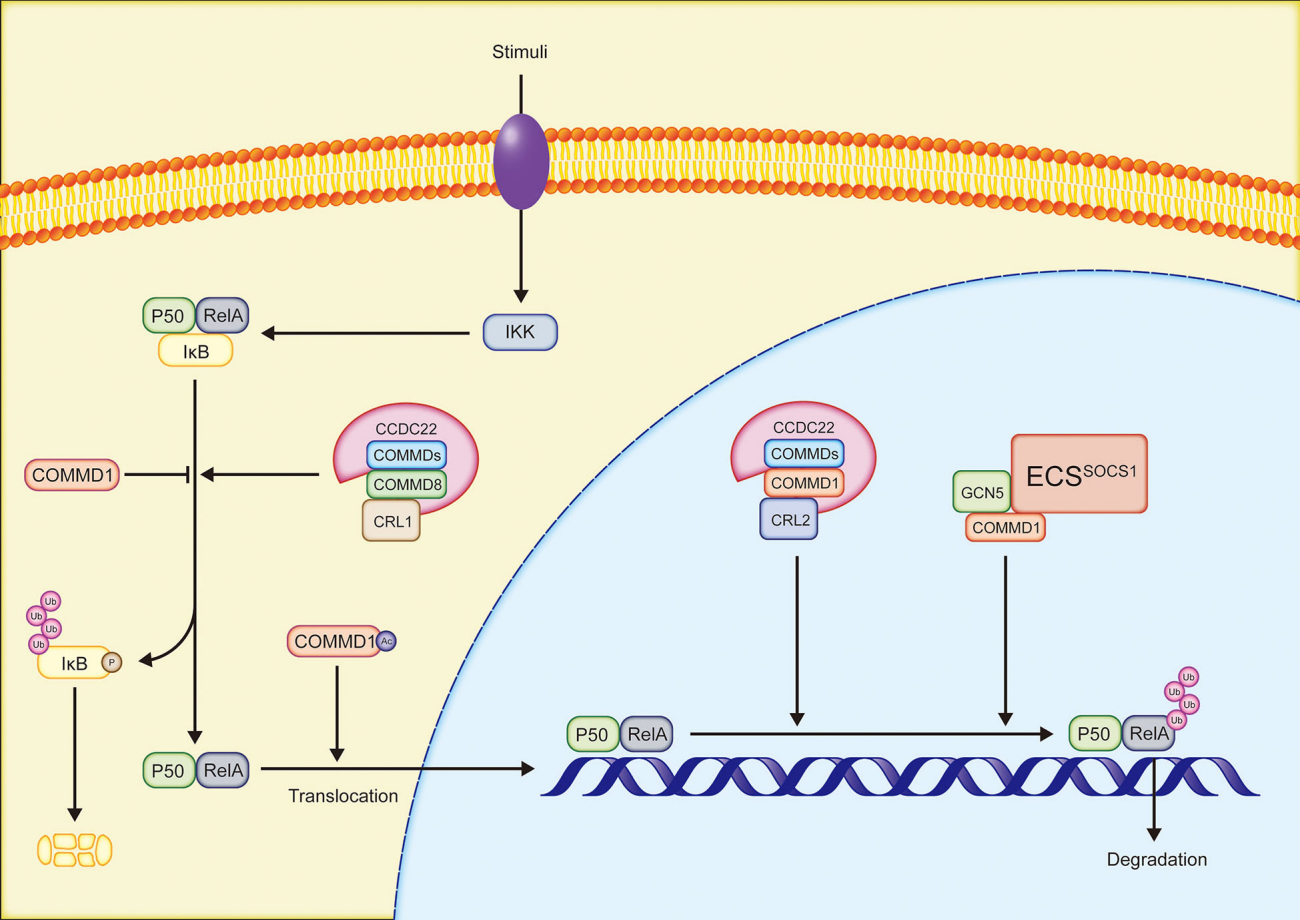
Simplified overview of the regulation of NF-κB pathway by COMMD proteins: COMMD, copper metabolism MURR1 domain-containing; CCDC22, coiled-coil domain-containing protein 22; CRL, Cullin-RING ligase; IKK, IκB kinase complex; GCN5, general control non-repressed protein 5.

The NF-κB family is an essential transcription factor that regulates the expression of several genes and participates in a variety of physiological processes, such as immune response, inflammation, and cell survival. RelA(p65), RelB, cREL, NF-κB1(p50/p105), and NF-κB2(p52/p100) occur as homodimers or heterodimers in cells as five members of the NF-κB family. Furthermore, the NF-κB family members in inactive status bind to inhibitor of NF-κB (IκB) (7, 50). In the canonical NF-κB pathway, PAMPs/DAMPs (such as LPS and CpG DNA), proinflammatory cytokines, and other stimulations, activate the receptors on the cell membrane. IκB is subsequently phosphorylated by IκB kinase (IKK) and degraded by proteasomes. RelA/p50 dimers that are no longer inhibited by IκB translocate to the nucleus to bind DNA and mediate transcription (50). In the majority of instances, all ten COMMD proteins may bind selectively to distinct subunits of NF-κB and inhibit the activity of NF-κB (6, 51)(**Figure 2**). COMMD1 is the most well investigated regulator of the NF-κB signaling pathway. COMMD1 interacts with ECSSOCS1, a multimeric E3 ubiquitin ligase complex composed of Elongins B and C, cullin2, and SOCS1, in order to increase RelA binding to SOCS1, resulting in the ubiquitination and proteasomal degradation of RelA, hence inhibiting NF-κB-mediated transcription (52). RelA Ser468 phosphorylation induced by IKK is required for COMMD1-dependent ubiquitination of RelA (53, 54). The phosphorylation of RelA at Ser468 increases its binding to general control non-repressed protein 5(GCN5), which forms a complex with COMMD1 and ECSSOCS1, increasing RelA ubiquitination and degradation (54). Notably, COMMD1 continues to occupy the promoter site even after RelA is removed, indicating that COMMD1 may inhibit the expression of NF-κB target genes by this method (53). In addition, COMMD1 overexpression inhibits NF-κB-mediated transcription by decreasing the binding time of RelA to chromosomes (6) and IκB ubiquitination (51, 55). Acetylated COMMD1 in the cytoplasm induced the nucleolus translocation of RelA to mediate apoptosis by ubiquitylating RelA (56). Acetylated COMMD1 ubiquitinates RelA. Ubiquitinated RelA translocates to the nucleus to mediate apoptosis. CCDC22, a highly conserved protein associated with X-linked intellectual disability, binds to COMMD proteins in conjunction to regulate the NF-κB signaling pathway. Cullin1 interacts with the CCDC22-COMMD8 complex to enhance IκB ubiquitination and NF-κB-mediated transcription. In contrast, the CCDC22-COMMD1 complex binds Cullin2 in order to promote RelA ubiquitination and inhibits the NF-κB pathway. Given that CCDC22 deficit inhibits NF-κB activation, the CCDC22-COMMD8 complex may serve as the primary regulator (57). Reportedly, COMMD7 enhances the activation of the NF-κB signaling pathway in HCC, which will be described in depth in the next section of this review (58).

### 2.5 Other biological functions of the COMMD protein family

Superoxide dismutase 1(SOD1), a member of the Cu,Zn superoxide dismutase family, catalyzes the production of molecular oxygen and hydrogen peroxide from superoxide anions, therefore acting as an antioxidant. Homodimer formation is the last stage in SOD1 maturation, and COMMD1 may decrease the amount of SOD1 homodimers, hence inhibiting SOD1 maturation and activity (59). Also regulated by COMMD1 is the hypoxia inducible factor 1 (HIF-1). Overexpression of *COMMD1* promotes the degradation of HIF-1α, while COMMD1 deficiency raises the stability of HIF-1α, increasing the transcription of HIF-1α target genes, and resulting in developmental defects and embryonic lethality (60). On the one hand, COMMD1 competitively inhibits Heat Shock Protein 90 (HSP90), which binds to and stabilizes HIF-1α. On the other hand, after binding to HIF-1α, COMMD1 cooperates with Heat Shock Protein 70(HSP70) to induce the ubiquitin-independent degradation of HIF-1 (61). In addition, COMMD1 may directly inhibit the formation of HIF-α and HIF-β to heterodimers with transcriptional activity (62). In a recent investigation on hepatocellular cancer, COMMD10 was also discovered to bind and inhibit HIF-1α (63), and it has to be determined if additional members of the COMMD protein family have comparable effects. Additionally, the COMMD prtotein family may engage in cell cycle control. Overexpression or silencing of *COMMD1* in HEK293T cells regulates the cell cycle and cell proliferation *via* altering the level of p21 Cip1 (64). The interaction between Lamin A and COMMD1 indicates that COMMD1 may potentially be associated with aging and laminopathies (65). Many investigations have shown that the COMMD protein family is broadly involved in physiological processes, including copper metabolism, endosomal sorting, ion transport, transcriptional control, and others; nevertheless, the probable molecular mechanism remains unexplored.

## 3 The COMMD protein family and tumors

### 3.1 Lung cancer

Lung cancer is a leading cause of cancer-related mortality, with roughly 85% of cases attributable to smoking (which is also affected by other environmental factors). Lung cancer is separated histologically into small cell lung cancer (SCLC) and non-small cell lung cancer (NSCLC); NSCLC includes adenocarcinoma (ADC), squamous cell carcinoma (SCC), and large cell carcinoma (undifferentiated NSCLC), accounting for over 80% of lung cancer cases and causing over 1.2 million deaths annually. Despite the widespread use of surgery, radiotherapy, chemotherapy, targeted therapy, and other treatments for the treatment of lung cancer, the prognosis of patients remains dismal, and the 5-year overall survival rate is just 15% (66, 67). By upregulating genes associated to cell proliferation, metastasis, angiogenesis, and anti-apoptosis, NF-κB may contribute to oncogenesis. It is believed that aberrant activation of the NF-κB signaling pathway promotes tumor survival and medication resistance by upregulating anti-apoptotic proteins and genes (67, 68). Given the obvious inhibitory effect of the COMMD protein family on NF-κB, it is plausible to hypothesize that COMMD proteins play a crucial role in oncogenesis, progression, and death. CIGB-552 is a synthetic anti-tumor peptide that stimulates the ubiquitination of RelA, a subunit of NF-κB, in lung cancer cells, therefore decreasing the anti-apoptotic action of NF-κB and promoting the death of tumor cells. The protein-protein interaction between COMMD1 and CIGB-552 and the accumulation of COMMD1 were also observed in lung cancer cells influenced by CIGB-552. Further research revealed that the *COMMD1* knockout inhibited CIGB-552-induced NF-κB degradation and cell death. Therefore, *COMMD1* upregulation produce antitumor effects through inhibiting the NF-κB signaling pathway (68). HIF-1 is another COMMD1-regulated transcription factor, and is involved in energy metabolism, cell growth, and angiogenesis. Similar to NF-κB, CIGB-552 promotes COMMD1 accumulation and inhibits HIF-1 activity in H460 cells (69), hence exerting anti-inflammatory and anti-angiogenic actions. CIGB-552 and cisplatin performed a synergistic impact in inhibiting tumor cell growth, inducing apoptosis and oxidative stress response, and overcoming cisplatin resistance in a recent research involving lung cancer cells and mice models (70). It is worth noting that COMMD1 promoted the repair of DNA double-strand breaks (DSBs) in lung cancer cells. The research also revealed that the upregulation of *COMMD1* increased the proliferation of NSCLC cells and was associated with a bad prognosis for patients. Even *COMMD1*-knockdown might increase the radiation sensitivity of NSCLC cells (17). As a potential novel anticancer treatment target, the function of COMMD1 in NSCLC requires additional investigation. In addition to COMMD1, additional COMMD protein family members have showed promise as prospective lung cancer therapeutic targets. Compared to healthy tissues and cells, the gene and protein expression levels of *COMMD4* is upregulated in NSCLC, and patients with high *COMMD4* expression are more likely to have a poor prognosis. *COMMD4* depletion significantly inhibits NSCLC cell growth, induces mitotic catastrophe and death, and increases NSCLC sensibility to irradiation and camptothecin. Because irradiation and camptothecin might produce DNA DSBs, COMMD4 activity is crucial for protecting NSCLC cells from DNA damage (18). COMMD8 is up-regulated in NSCLC, and COMMD8 promoted cell proliferation, migration, glycolysis and inhibited cell apoptosis in NSCLC. In addition, research have shown that COMMD8 might be activated in NSCLC through activating MALAT1/miR-613 axis and LINC00657/miR-26b-5p axis, playing a carcinogenic role (19, 71). In NSCLC cells, DRTF1 and E2F1 join to create a heterodimer in order to enhance G1/S transition and inhibit P53 activity. The high expression of *COMMD9* in NSCLC increases cell proliferation and invasion. *COMMD9*-knockdown results in G1/S arrest, induces autophagy, inhibits the activation of TFDP1/E2F1 and enhances P53 signaling pathway, playing an antitumor effect (20).

### 3.2 Hepatocellular carcinoma

Hepatocellular carcinoma (HCC) is the most prevalent type of primary liver cancer and the third biggest cause of cancer-related mortality globally. In recent years, several studies have shown that the COMMD protein family plays a crucial role in the development and incidence of HCC. Analysis of bioinformatic data revealed that *COMMD2* was highly expressed in HCC and promoted the proliferation and migration of HCC cells, making it a potential oncogene. A high expression of *COMMD2* is associated with poor prognosis, higher histological grade, more advanced clinical stage, lymph node metastasis and the TP53 mutation status in HCC patients. Additionally, COMMD2 impacts the overall survival of patients with HCC by enhancing tumor immune infiltration (21). Similarly with *COMMD2*, *COMMD3*, *COMMD7*, and *COMMD8* are overexpressed in HCC, increasing cell proliferation, migration, and invasion (22, 24, 58). In HCC patients, *COMMD3* upregulation is related with advanced TNM stage, poor overall survival, and vascular invasion. In null mice with subcutaneous xenograft tumors, silencing *COMMD3* inhibited tumor development and expression of HIF1α, VEGF, and CD34. In HCC, COMMD3 seemed to operate as an upstream regulator of HIF1α (22). Several investigations have elaborated on the involvement of COMMD7 in HCC, despite the fact that COMMD7 inhibits NF-κB activation in HEK293T cells (72). Instead, COMMD7 stimulated NF-κB in HCC cells. A positive feedback loop was formed when *COMMD7* silencing induces HCC cell apoptosis by inhibiting NF-κB, and inhibited NF-κB further lowerd *COMMD7* transcription. Researchers suggested that the inconsistent action of COMMD7 on NF-κB might be due to the different cell types or cytokine stimulation (73, 74). *COMMD7* overexpression alone activated NF-κB signaling pathway by increasing PIAS4-mediated NEMO sumoylation in Nanog^+^ HCSCs, but the overexpression of *COMMD1* and *COMMD7* together ended NF-κB signaling (58). Moreover, overexpression of *COMMD7* in HCC might increase cell proliferation and invasion by promoting ROS accumulation and activating NF-κB, and downstream CXCL10 (23, 75). COMMD10, unlike COMMD7, inhibits HCC cell proliferation by inhibits the NF-κB signaling pathway and enhances cell death by activating the signaling pathway of Bcl‐2/Bax/caspase-9/3. On the on hand, COMMD10 binds to the Rel homology domain of p65 in HCC cells and inhibits the nuclear translocation of NF-κB. On the other hand, COMMD10 reduces the ubiquitination and degradation of IκB, hence reducing the action of NF-κB (25). Another research revealed that COMMD10 increased radiosensitivity in HCC cells by decreasing intracellular Cu, inhibiting HIF1α, and stimulating ferroptosis (63). COMMD10 exhibits an anticancer impact in HCC and is related with increased survival.

### 3.3 Other cancers

Similar to the effect in lung cancer, COMMD1 exerts an anti-tumor effect in malignant tumors, including prostate cancer (76), diffuse large B-cell lymphoma (77), head and neck squamous-cell carcinoma (HNSCC) (78), and neuroblastoma (79). Downregulation of *COMMD1* was related with a poor prognosis for diffuse large B-cell lymphoma, according to bioinformatics study (77). COMMD1 decreases tumor cell activity by inhibiting the NF-κB signaling pathway in prostate cancer and neuroblastoma (76, 79). COMMD1 increases its stability by forming a complex with DRR1 and F-actin in the nucleus of neuroblastoma. Subsequently, DRR1 and COMMD1 inhibits cyclinD1 expression, the G1/S transition, and neuroblastoma cell proliferation (79). miR-205 enhances inflammatory and stemness properties in stemness-enriched HNSCC cells and promotes tumorigenesis and tumor progression *via* downregulating *COMMD1*. *COMMD1* downregulation also enhances the activation of NF-κB in HNSCC cells, which in turn promotes the release of inflammatory factors into the tumor microenvironment and further increases miR-205 expression (78). HIF-1, a transcription factor, is similarly regulated by COMMD1 in tumor cells. In HT29 and U2OS cells, reduced *COMMD1* expression inhibits many HIF-responsive genes, including *VEGFA*, *TGFA*, *HK2*, and *GLUT1*. Notably, *COMMD1*-deficient tumor cells did not exhibit an increase of protein level in HIF-1α or HIF-2α. Further research demonstrated that COMMD1 bond to HIF-1α in a competitive manner and prevented the formation of the HIF-1α/β heterodimer (62), hence inhibiting the DNA binding and transcriptional activity of HIF-1α. Furthermore, COMMD1 is associated to drug resistance in ovarian cancer and multiple myeloma (26, 80). Ovarian cancer cells A2780 are more susceptible to cisplatin when nuclear *COMMD1* expression is increased. The mechanism might include COMMD1’s regulation of the G2/M checkpoint, DNA repair, and apoptosis (26). In Bortezomib-resistant multiple myeloma, however, *COMMD1* expression was shown to be elevated. By inhibiting SCF complex, *COMMD1*-knockdown could overcome Bortezomib resistance (80). By activating COMMD1, CIGB-552 reduced the growth of breast cancer cells MCF-7 and colon cancer cells HT-29 in addition to lung cancer cells (81). Gene rearrangement is often linked with an aggressive phenotype and poor prognosis in prostate cancer. The researchers discovered that *COMMD3: BMI1* fusion expression was significantly elevated in metastatic prostate cancer. COMMD3 activates oncogenes such as c-MYC, which promotes prostate cancer cell proliferation, migration, and invasion; COMMD3 expression is positively connected with tumor recurrence and decreased survival rate (82). COMMD5, also known as hypertension-related, calcium-regulated gene (HCaRG), is strongly expressed in renal proximal tubules, where it regulates cell proliferation and differentiation, and accelerate the repair of renal tubules. However, excessive proliferation and poor differentiation are significant tumor progression indicators. In renal cancer cells, COMMD5 decreases proliferation, improves differentiation, accelerates autophagic cell death, and lowers VEGF production through downregulating HIF-1α, ultimately inhibiting tumor angiogenesis. The overexpression of ErbB receptors (including EGFR, ErbB2, ErbB3, and other receptors) is related with the development and progression of cancer, and have been observed in several epithelial-derived human malignancies. In renal cell cancer, COMMD5 inhibits the methylation of EGFR and ErbB3 promoters. COMMD5 significantly reduces the transcription and translation of EGFR and ErbB3 by inhibiting the methylation of EGFR and ErbB3 promoters, ultimately performing an antitumor function (83, 84). Expression of *COMMD5* is reduced in gastric cancer. Rosiglitazone, a synthetic PPAR agonist, was associated by *COMMD5* up-regulation in inhibiting gastric carcinogenesis, according to studies. However, the precise function of COMMD5 in gastric cancer requires additional investigation (85). In pancreatic ductal adenocarcinoma (PDAC) (86) and acute myeloid leukemia (87), high *COMMD7* expression is associated with a poor prognosis. COMMD7 is positively linked with PDAC histological differentiation, lymph node metastasis, and TNM staging. By downregulating cyclin D1, inhibiting MMP-2 secretion, and activating the ERK1/2 and apoptosis signaling pathways, inhibition of COMMD7 may reduce tumor growth and invasion (86). Additionally, COMMD7 is connected with medication resistance. In gastric cancer, the activation of the Linc00852/miR-514a-5p/COMMD7 axis enhances cisplatin resistance (88). Loss of ETV6 function is associated with the development of hematological cancers. Using a functional genome-wide shRNA screen, researchers discovered that *COMMD9*-knockdown in leukemic cells might inhibit *ETV6* transcriptional activity (89). However, the underlying mechanism by which COMMD9 regulates ETV6 remains unknown. Formin-like2 (FMNL2) is a member of the Formins family, which is upregulated in colorectal cancer and increases tumor cell motility, invasion, and metastasis (90). Additional research demonstrated that FMNL2 enhanced the ubiquitin-mediated proteasome degradation of COMMD10, hence reducing the nuclear translocation of NF-κB subunit p65 and promoting tumor progression. *COMMD10* downregulation is often correlated with worse clinical outcomes. Consequently, *COMMD10* may be employed as an independent survival predictive marker for CRC patients (91). Bioinformatics analysis revealed that the expression of *COMMD10* in lung squamous cell carcinoma, breast invasive carcinoma, and renal clear cell carcinoma is distinct from that of their corresponding normal tissues. Moreover, high *COMMD10* expression in renal clear cell carcinoma is predictive with improved patient survival (27).

## 4 Conclusions

COMMD proteins serve various biological functions in eucaryon. It is hypothesized that the action of COMMD1 on copper metabolism is connected to its regulation of the stability as well as intracellular transport of copper-transporting ATPases Atp7a and Atp7b (31, 33, 35). In addition, COMMD1 may directly bind to Cu (II), although the importance of this binding for copper metabolism regulation is unknown (36). In the hepatocyte COMMD-specific knockout mouse model, the absence of COMMD1, COMMD6, or COMMD9 similarly impaired the endosomal recycling of Atp7b, according to a research published last year (37). However, the regulation on copper metabolism by COMMD protein family members other than COMMD1 remains poorly known. COMMD proteins may also form CCC complexes with CCDC22, CCDC93, and C16orf62, and participate in endosomal sorting of many transmembrane proteins including Atp7a (35), LDLR (41), and α5β1-integrin (43). It has to be determined if the COMMD protein family and CCC complex are involved in the protein trafficking of other proteins. COMMD may also regulate ion transport by influencing the ubiquitination and surface expression of ENaC (45), CFTR (48), and NKCC1 (92) on the cell membrane. COMMD1 increases the ubiquitination of ENaC (45), CFTR (48), NKCC1 (92). COMMD1 promotes the ubiquitination of ENaC (45), NKCC1 (92) and Nf-κB (52), but inhibits the ubiquitination of CFTR (48) and IκB (51).Therefore, the precise mechanism of COMMD protein-mediated ubiquitination needs further investigation. In addition, the action of COMMD proteins in regulating NF-κB, HIF, and other transcription factors enables it to play a crucial role in inflammation, cancer, and other disease progression.

Almost all COMMD proteins are implicated in regulating cancer signaling (**Table 1**). They regulate the proliferation, differentiation, invasion, and influence metastasis of tumor cells, tumor angiogenesis, chemotherapeutic drug resistance, and prognosis of cancer. Current research indicates that the great majority of COMMD proteins, such as COMMD2 (21), COMMD3 (22), COMMD4 (18), COMMD7 (7), COMMD8 (24) and COMMD9 (20), are up-regulated in tumor cells and promote tumor proliferation, invasion, and metastasis. COMMD1 (68, 76), COMMD5 (83), and COMMD10 (25, 91) have antitumor effects in general. In a recent study, researchers used the GEPIA database to evaluate *COMMD6* mRNA expression in 31 human cancers with matching normal tissue species. *COMMD6* was discovered to be highly expressed in 20 types of tumors, such as colon cancer and brain lower grade glioma (LGG), and to be poorly expressed in 11 other types of tumors, including adrenocortical carcinoma (ACC) and pheochromocytoma. Further research indicated that high expression of *COMMD6* is associated with shorter survival in HNSC, cholangiocarcinoma, and ACC patients, but it correlates with longer survival in LGG, uveal melanoma, testicular germ cell tumors, thyroid carcinoma, and uterine corpus endometrial carcinoma patients (93). Depending on the kind of COMMD protein and the type of tumor, it is evident that the function of COMMD protein differs somewhat. Even if there are only dozens of pieces of experimental researches on the function of COMMD protein in tumors, we can still determine the general rule regarding the involvement of COMMD protein in tumors from these restricted data. Often, the same COMMD protein has a similar role in various tumors. For instance, COMMD1 inhibits the development of cancer in a number of malignancies. Inhibition of COMMD1 expression stimulated the growth of tumor cells in lung cancer (69), neuroblastoma (79), head and neck squamous cell carcinoma (78), and prostate cancer (76). The mechanism may be associated with COMMD1’s deactivation of HIF-1 and NF-κB. In contrast, the high expression of COMMD7 stimulated the proliferation and invasion of hepatocellular carcinoma (23) and pancreatic ductal adenocarcinoma (86). Various COMMD proteins serve different roles for the same tumor. COMMD1 (68) functioned as an anti-cancer agent in lung cancer, whereas COMMD4 (18), COMMD8 (19), and COMMD9 (20) increased the growth of lung cancer cells. Due to a dearth of relevant research, it remains unclear if distinct COMMD proteins have synergistic or antagonistic effects on the same tumor. Recent research has shown the relationship between COMMD1 and COMMD7. Overexpression of COMMD1 and COMMD7 concurrently inhibited NF-κB signaling in Nanog+ HCSCs (58). It is hypothesized that the antagonistic action of COMMD1 on COMMD7 is achieved through regulating the NF-κB signaling pathway. However, it remains unknown if additional COMMD proteins interact with each other in tumor cells. In terms of molecular mechanism, COMMD proteins in tumor cells regulate the oncogenes expression of NF-κB, HIF (68), C-YMC (82), and others. In lung cancer (68), prostate cancer (76), and neuroblastoma (79), for example, COMMD1 exerts anti-tumor actions *via* inhibiting NF-κB. Further investigation is required to determine if additional oncogenes are regulated by COMMD proteins. In addition, COMMD proteins are engaged in the regulation of cell cycle (20) in tumors and is strongly associated with apoptosis (18), autophagy, ferroptosis (63) and other signaling pathways, influencing the proliferation and death of tumor cells. Given the broad significance of COMMD proteins in tumor-related signaling pathways, COMMD is a viable therapeutic target for tumors. CIGB-552 is a synthetic anti-tumor peptide that functions as an anti-tumor agent in lung cancer and colorectal cancer by activating COMMD1 (69, 94). Several noncoding RNAs, such as Linc00852 (88), Linc657 (71), MALAT1 (19), and MNX1-AS1 (24), have been reported to influence the incidence, development of tumor, and the death of tumor cells by influencing COMMD proteins expression. Prospects for the discovery of novel anticancer medicines that target the COMMD proteins and their upstream pathways are vast. In addition, since COMMD proteins are expressed in a range of tumor tissues and there are considerable variations between normal and malignant tissues, COMMD has high predictive value that may be used for clinical staging and prognostic assessment of cancers.

**Table 1:** The roles of COMMD proteins in different tumors.

Type of cancer	Type of COMMD proteins	Functions
Lung cancer	COMMD1	COMMD1 inhibits Nf-κb and HIF-1 in H460 cells, and plays an anti-inflammatory, anti-tumor and anti-angiogenesis role (68, 69). *COMMD1* is highly expressed in NSCLC cells and repairs DNA DSBs. Up-regulation of *COMMD1* is associated with poor prognosis in patients with NSCLC (17).
Lung cancer	COMMD4	*COMMD4* depletion significantly inhibits NSCLC cell proliferation, induces mitotic catastrophe and apoptosis, and increases the sensitivity of NSCLC to irradiation and camptothecin (18)
Lung cancer	COMMD8	*COMMD8* expression is up-regulated in NSCLC; COMMD8 promotes cell proliferation, migration, glycolysis, and inhibits apoptosis (19, 71)
Lung cancer	COMMD9	*COMMD9* is highly expressed in NSCLC; COMMD9 promotes cell proliferation, migration, cell cycle G1/S transition, inhibits autophagy, promotes *TFDP1/E2F1* transcription activity and inhibits P53 signaling pathway (20)
Hepatocellular carcinoma	COMMD2	*COMMD2* is highly expressed in HCC, and COMMD2 promotes the proliferation, migration and tumor immune infiltration of HCC cells, which is associated with poor prognosis, higher histological grade, more advanced clinical stage, lymph node metastasis and the TP53 mutation status in HCC patients (21).
Hepatocellular carcinoma	COMMD3	*COMMD3* is highly expressed in HCC; COMMD3 promotes the growth, migration, invasion and angiogenesis of HCC, and is associated with advanced TNM staging, poor overall survival and vascular invasion of HCC patients (22)
Hepatocellular carcinoma	COMMD7	*COMMD7* is highly expressed in HCC; COMMD7 promotes HCC proliferation, migration, invasion, activation of Nf-κB signaling pathway and CXCL10 (23, 73–75).
Hepatocellular carcinoma	COMMD8	*COMMD8* is highly expressed in HCC, and COMMD8 promotes the proliferation, migration and invasion of HCC (24)
Hepatocellular carcinoma	COMMD10	*COMMD10* is poorly expressed in HCC; COMMD10 inhibits HCC proliferation, enhances apoptosis, ferroptosis, and radiosensitivity, and inhibits Nf-κb and HIF1α signaling pathways; COMMD10 is associated with longer overall survival (25, 63).
Prostate cancer	COMMD1	sCLU promotes COMMD1 and I-κB degradation, enhances Nf-κb activity and tumor cell survival in prostate cancer cells (76).
Prostate cancer	COMMD3	*COMMD3: BMI1* fusion expression and COMMD3 protein increases significantly in metastatic prostate cancer; COMMD3 activates oncogenes such as c-MYC, promotes prostate proliferation, migration and invasion; Increased *COMMD3* expression is positively correlated with tumor recurrence and reduced survival (82).
Diffuse large B-cell lymphoma	COMMD1	Down-regulation of *COMMD1* is associated with poor prognosis in diffuse large B-cell lymphoma (77).
Head and neck squamous-cell carcinoma	COMMD1	*COMMD1* is poorly expressed in HNSCC. miR-205-mediated down-regulation of *COMMD1* increases inflammatory and stemness features in stemness-enriched cancer cells, and promotes tumorigenesis and tumor growth (78)
Neuroblastoma	COMMD1	DRR1, F-actin and COMMD1 form a complex in the nucleus, and the stability of COMMD1 in the complex is enhanced to promote the degradation of Nf-κB; DRR1 and COMMD1 inhibit cyclinD1 expression, G1/S transition and cell proliferation in neuroblastoma (79).
Ovarian cancer	COMMD1	Increased nuclear *COMMD1* expression helps overcome Cisplatin resistance in ovarian cancer cells A2780 (26)
Multiple myeloma	COMMD1	High expression of *COMMD1* is associated with Bortezomib resistance in multiple myeloma (80)
Renal cell carcinoma	COMMD5	*COMMD5* is poorly expressed in renal cell carcinoma; COMMD5 inhibits renal cell carcinoma proliferation, promotes differentiation, improves prognosis, enhances autophagic cell death, and inhibits tumor angiogenesis; The antitumor effect of COMMD5 is related to the inhibition of EGFR, ErbB receptors and VEGF (83, 84).
Gastric cancer	COMMD5	*COMMD5* is poorly expressed in gastric cancer. Rosiglitazone, a synthetic PPARγ agonist, is associated with COMMD5 up-regulation in the inhibition of gastric carcinogenesis (85)
Gastric cancer	COMMD7	COMMD7 is associated with cisplatin resistance in gastric cancer (88).
Pancreatic ductal adenocarcinoma	COMMD7	*COMMD7* is highly expressed in PDAC; COMMD7 is positively correlated with PDAC histological differentiation, lymph node metastasis and TMN stage. Inhibition of COMMD7 may inhibit tumor proliferation and invasion by down-regulating cyclin D1, inhibiting MMP-2 secretion, and activating ERK1/2 and apoptosis (86)
Acute myeloid leukemia	COMMD7	High expression of *COMMD7* is associated with poor prognosis in acute myeloid leukemia (87)
Colorectal cancer	COMMD10	*COMMD10* is downregulated in colorectal cancer; COMMD10 inhibits colorectal cancer invasion and metastasis by inhibiting nuclear accumulation of p65 (91).
Renal clear cell carcinoma	COMMD10	*COMMD10* is highly expressed in renal clear cell carcinoma and predicts better overall survival of patients (27).

Although many pieces of research have established that the COMMD proteins are closely associated with cancer, the actual molecular mechanism is still not well understood, and the great majority of studies have focused on the COMMD as a regulation for oncogenes of NF-κB and HIF. Copper homeostasis may be employed as a novel cancer therapeutic target, according to studies. Cu levels are greatly elevated in several malignant tumors, such as breast cancer, ovarian cancer, and gastric cancer. Cu can also enhance cancer proliferation, angiogenesis, and metastasis (28). Although COMMD proteins play a critical role in the regulation of copper metabolism, few studies have shown that interference with copper homeostasis by COMMD proteins affect tumor cell activity. Cu improved the radioresistance of HCC cells in a recent research, but COMMD10 increased their radiosensitivity by decreasing intracellular copper levels and inducing ferroptosis (63). This discovery offers fresh insights into the relationship between the COMMD protein family and tumors. In addition, mounting data demonstrates that ENaC (95), CFTR (96), SOD1 (97) and other signaling molecules play crucial roles in tumor cell proliferation, migration, and apoptosis, and this review notes that these molecules are similarly regulated by COMMD proteins. To be investigated further is the existence of a complex protein-protein interaction network between COMMD proteins and tumor phenotypes.

In conclusion, the COMMD proteins family has a broad variety of biological roles and is involved in tumor proliferation, invasion, metastasis, angiogenesis, and drug resistance, among other functions. Additionally, the COMMD protein may be exploited as a therapeutic target and a biomarker for the prognosis and staging of malignancies. Additional research on the function of the COMMD proteins in cancer is beneficial for the clinical diagnosis and treatment of malignancies.

## Author contributions

GY drafted the manuscript. CZ, LW, HF, ZL, XZ and XY discussed and revised the manuscript. All authors contributed to the article and approved the submitted version.
